# Germacrene D, A Common Sesquiterpene in the Genus *Bursera* (Burseraceae)

**DOI:** 10.3390/molecules14125289

**Published:** 2009-12-15

**Authors:** Koji Noge, Judith X. Becerra

**Affiliations:** 1 Department of Entomology, University of Arizona, Tucson, AZ 85721, USA; 2 Department of Biosphere 2, University of Arizona, Tucson, AZ 85721, USA; E-Mail: jxb@email.arizona.edu (J.X.B.)

**Keywords:** *Bursera*, burseraceae, germacrene D, monoterpenes, sesquiterpenes

## Abstract

The volatile components of the leaves of five *Bursera* species, *B*. *copallifera*, *B*. *exselsa*, *B*. *mirandae*, *B*. *ruticola* and *B*. *fagaroides* var. *purpusii* were determined by gas chromatography–mass spectrometry (GC–MS). Germacrene D was one of the predominant components (15.1–56.2%) of all of these species. Germacrene D has also been found in other *Bursera* species and some species of *Commiphora*, the sister group of *Bursera*, suggesting that the production of germacrene D might be an ancient trait in the genus *Bursera*.

## 1. Introduction

The genus *Bursera* (Burseraceae) comprises approximately 100 species with a geographical distribution extending from the southwestern United States to Peru. The genus predominates in the tropical dry forests of Mexico where about 85 species coexist and about 75 of them are endemic [1,2,3]. The genus *Bursera* is divided into two sections, *Bullockia* and *Bursera*. *Bursera* release their resins when they are attacked by their herbivore, *Blepharida* beetles (Chrysomelidae: Alticinae) [4,5]. The resin is reported to decrease the survival and growth of *Blepharida* beetles [5,6]. The chemical analysis of the leaf components of *B*. *biflora* and *B*. *schlechtendalii* revealed that their resins consisted mostly of mono- and sesquiterpenes, and alkanes [7,8].

The volatile chemistry of five species, *B*. *copallifera*, *B*. *excelsa*, *B*. *mirandae*, *B*. *ruticola* (belonging to section *Bullockia*) and *B*. *fagaroides* var. *purpusii* (from section *Bursera*), were analyzed in this study. *Bursera copallifera* is native to the dry forests from the state of Nayarit to Oaxaca and Puebla at altitudes of between 1,000 and 1,900 m. *Bursera excelsa* is mostly found along the Pacific coast at low altitudes, while *B*. *mirandae* has a narrow distribution in the north of the states of Guerrero and Oaxaca. *Bursera ruticola* is a narrow endemic form the tip of Baja California. *Bursera fagaroides* var. *purpusii* is an abundant species also of relative wide distribution in the South of Mexico at altitudes between 300 and 900 m. All of these species are abundant either locally or regionally. Yet, little information is known about their chemistry.

## 2. Results and Discussion

The components found in the five *Bursera* species analyzed are summarized in Table 1. Since the chemical profiles of the field samples were very similar to those of the greenhouse plants, only the latter ones are presented. The similarity of the chemical profiles between field and greenhouse samples suggests that the production of these compounds is quite stable even in the face of environmental changes. The chemical profiles of each one of these species were species-specific. For example, some compounds such as α-thujene, α-phellandrene, β-ocimene, and others were detected in only one of the species. Some compounds such as α-pinene, β-caryophyllene, germacrene D and bicyclogermacrene, however, were detected in all of the species examined as either major (higher than 10% of total) or minor (less than 10% of total) components. The ratio of monoterpenes to sesquiterpenes was also different among the species (Table 1). The composition pattern could be classified into three types; (1) monoterpene-rich group (*B*. *fagaroides* var. *purpusii*), (2) sesquiterpene-rich group (*B*. *copallifera* and *B*. *excelsa*), and (3) the intermediate group (both monoterpene- and sesquiterpene-rich; *B*. *mirandae* and *B*. *ruticola*). It seems that terpene production in the species of the section *Bursera* tends to be biased toward monoterpenes, and a high percentage of these compounds have been found in the leaves of other species belonging to this section such as *B*. *lancifolia*, *B*. *rezedowskii*, *B*. *schlechtendalii* and *B*. *morelensis* (75–95%) [6]. 

Germacrene D was one of the predominant components found in the leaves of the five species analyzed in this study (15.1–56.2%; Table 1). The concentration of germacrene D was 0.14 ± 0.02 mg per gram of fresh leaves (mg/g.l., means ± SD, N = 5) in *B*. *copallifera*, 0.14 ± 0.03 mg/g.l. in *B*. *excelsa*, 0.12 ± 0.03 mg/g.l. in *B*. *mirandae*, 1.06 ± 0.33 mg/g.l. in *B*. *ruticola* and 0.13 ± 0.06 mg/g.l. in *B*. *fagaroides* var. *purpusii*, respectively. A preliminary chemical screening of the leaves of 26 other *Bursera* species also revealed the presence of germacrene D in all of them (2.6–53.8%, data not shown), suggesting that this compound is common in the genus. Germacrene D has been recently reported from the leaves of *B*. *simaruba* [9] and the stem of *B*. *graveolens* [10], respectively. Germacrene D has also been found in some species of *Commiphora*, the sister group of *Bursera*, such as *C. africana* (Noge and Becerra, unpublished), *C*. *holtziana* [11] and *C*. *myrrha* [12]. Thus, producing germacrene D might be an ancient trait in the genus *Bursera*.

**Table 1:** Composition in the leaf of five *Bursera* species.

Compound	*t*_R_ (min)*^a^*	ID *^b^*	Composition (%) *^c^*				
			*B*. *copallifera*	*B*. *excelsa*	*B*. *mirandae*	*B*. *ruticola*	*B*. *fagaroides* var. *purpusii*
α-Thujene	7.65	2	0.2	–	–	5.3	–
α-Pinene	7.81	1	0.7	1.6	6.6	10.3	67.8
Camphene	8.15	2	–	–	–	1.2	1.2
Sabinene	8.53	2	–	–	–	2.8	1.2
β-Pinene	8.65	1	–	4.7	–	21.9	5.7
β-Myrcene	8.76	1	–	–	0.5	–	2.0
α-Phellandrene	9.13	1	0.3	1.2	15.0	–	–
*p*-Cymene	9.44	1	–	–	0.6	–	–
Limonene	9.52	1	–	–	–	0.4	0.9
β-Phellandrene	9.56	1	–	–	1.9	2.0	0.2
β-Ocimene	9.75	1	–	4.9	–	–	–
Sesquiterpene	14.21	3	–	2.6	–	–	–
α-Copaene	14.80	1	1.7	–	2.7	–	–
Sesquiterpene	14.94	3	1.7	6.4	0.7	0.5	–
β-Caryophyllene	15.43	1	9.6	15.0	14.4	18.3	4.3
α-Humulene	15.88	1	12.5	0.7	0.5	0.6	–
Sesquiterpene	15.93	3	0.4	–	0.5	–	–
Sesquiterpene	16.05	3	1.5	0.7	–	–	–
Germacrene D	16.18	1	56.2	50.5	36.6	31.9	15.1
Bicyclogermacrene	16.36	2	6.2	8.8	1.2	0.7	0.8
Sesquiterpene	16.55	3	2.4	–	–	1.2	–
Sesquiterpene	16.57	3	–	1.5	2.7	–	–
Sesquiterpene	17.33	3	1.0	1.3	–	0.6	–
Sesquiterpene	18.06	3	1.4	–	–	0.5	–
Sesquiterpene	18.27	3	–	–	–	1.0	–
Unknown	25.95	4	–	–	2.6	–	–
Unknown	26.58	4	–	–	–	0.9	–
Monoterpenes			1.2	12.4	24.6	43.9	79.0
Sesquiterpenes			94.6	87.5	59.3	55.3	20.2

*^a^* Retention times are based on GC-FID analysis with DB-5MS capillary column; *^b^* Method of identification: 1, matching GC retention time and mass spectrum with an authentic standard; 2, mass spectral matching with a library spectrum; 3, interpretation of the mass spectrum; 4, unidentified; *^c^* Percentages are based on GC peak area. Percentages higher than 10% are bolded. –, Not detected.

Germacrene D has been found not only in angiosperms and gymnosperms but also in bryophites (Table 2), yet despite its wide distribution, its biological function in plants is still not well understood. It has been proposed that germacrene D plays a role as a precursor of various sesquiterpenes such as cadinenes and selinenes [13,14]. Plant terpenes have often been reported as anti-herbivore defenses [15]. It has also been suggested that germacrene D, by itself may have deterrent effects against herbivores and it has been reported to have insecticidal activity against mosquitoes [16], as well as repellent activity against aphids [17] and ticks [11].

*Bursera* is attacked by a group of specialized chrysomelid beetles, the genus *Blepharida*. These beetles show a preference for colonizing chemically similar plants that are not necessarily phylogenetically close [18]. This preference for chemically similar plants might impose pressures on plants to develop divergent chemical components. Thus, the common presence of germacrene D in *Bursera* may be advantageous as a source of other terpenes that could make the plants chemically different and thus help them escape from herbivory by unadapted *Blepharida* beetles. We are currently investigating its potential role against *Bursera*’s herbivores. Being such an abundant and frequent compound in this genus, it is likely to play an interesting role in the functioning of these plants.

**Table 2:** Examples of the presence of germacrene D in plants.

Plant group	Plant species	Family	Reference
Spermatophytes			
Angiosperms			
Eudicots	*Bursera copallifera*	Burseraceae	This study
	*Bursera excelsa*	Burseraceae	This study
	*Bursera mirandae*	Burseraceae	This study
	*Bursera ruticola*	Burseraceae	This study
	*Bursera fagaroides* var. *purpusii*	Burseraceae	This study
	*Bursera graveolens*	Burseraceae	[10]
	*Bursera simaruba*	Burseraceae	[9]
	*Boswellia sacra*	Burseraceae	[19]
	*Commiphora Africana*	Burseraceae	Noge and Becerra, Unpublished
	*Commiphora holtziana*	Burseraceae	[11]
	*Commiphora myrrha*	Burseraceae	[12]
	*Protium icicariba*	Burseraceae	[20]
	*Eucalyptus dunnii*	Myrtaceae	[21]
	*Eugenia uniflora*	Myrtaceae	[22]
	*Citrus natsudaidai*	Rutaceae	[23]
	*Chloroxylon swieetenia*	Rutaceae	[16]
	*Zanthoxylum rhoifolium*	Rutaceae	[24]
	*Zanthoxylum setulosum*	Rutaceae	[24]
	*Angelica glauca*	Apiaceae	[25]
	*Torilis japonica*	Apiaceae	[26]
	*Altemisia annua*	Asteraceae	[27]
	*Brickellia veronicaefolia*	Asteraceae	[28]
	*Solidago altissima*	Asteraceae	[29]
	*Solidago Canadensis*	Asteraceae	[30]
	*Hemizygia petiolata*	Lamiaceae	[17]
	*Phlomis chimerae*	Lamiaceae	[31]
	*Phlomis grandiflora* var. *grandiflora*	Lamiaceae	[31]
	*Phlomis leucophracta*	Lamiaceae	[31]
	*Stachys germanica*	Lamiaceae	[32]
	*Stachys iva*	Lamiaceae	[32]
	*Lycopersicon hirsutum*	Solanaceae	[33]
Monocots	*Amomum subulatum*	Zingiberaceae	[34]
	*Curcuma rhizomes*	Zingiberaceae	[35]
Magnoliids	*Cananga odorata*	Annonaceae	[36]
	*Cryptocarya mandioccana*	Lauraceae	[14]
	*Talauma ovata*	Magnoliaceae	[37]
Gymnosperms	*Piper lanceaefolium*	Piperaceae	[38]
	*Araucaria bidwillii*	Araucariaceae	[39]
	*Araucaria heterophylla*	Araucariaceae	[39]
	*Pinus radiate*	Pinaceae	[40]
	*Halocarpus bidwillii*	Podocarpaceae	[41]
	*Podcarpus spicatus*	Podocarpaceae	[42]
Bryophytes	*Preissia quadrata*	Marchantiaceae	[43]

The genus *Bursera* is one of the dominant members of the tropical dry forests of Mexico in terms of both diversity and abundance. This study is a contribution to the chemical knowledge of this important group of plants. 

## 3. Experimental

### 3.1. Plant materials

Samples of leaves of one to five mature individuals from each species were collected from natural field populations and immediately extracted in dichloromethane. We also collected cuttings of each species and rooted them in a mixture of 70% pumice and 30% topsoil. All cuttings took and were kept in a greenhouse at the University of Arizona under similar environmental conditions to those in the field. When these cuttings developed leaves, they were also extracted for chemical analysis. Voucher specimens of all except for *B*. *ruticola* are deposited at the University of Arizona Herbarium (ARIZ) and the Mexican National Herbarium at the Autonomous National University of Mexico (MEXU) [2]. Voucher specimen of *B*. *ruticola* is deposited at ARIZ [3].

### 3.2. Sample preparation

Fresh leaf materials were collected from greenhouse plants (*B*. *copallifera*, 47–64 mg; *B*. *excelsa*, 42–141 mg; *B*. *mirandae*, 23–33 mg; *B*. *ruticola*, 35–74 mg; *B*. *fagaroides* var. *purpusii*, 35–52 mg) and immediately extracted for 24 h at 4 °C with 1 ml of dichloromethane containing 10 ng/μl anisole as an internal standard. The extracts were then collected into a new glass vial and kept at 4 °C until chemical analysis.

### 3.3. Chemical analysis

One microliter of each extract was analyzed by GC–MS (an Agilent 6890N gas chromatograph linked to an Agilent 5975B mass spectrometer, operated at 70 eV, with a HP-5MS capillary column, 30 m × 0.25 mm i.d., 0.25 μm in film thickness) and GC (the same gas chromatograph as GC–MS with a flame ionization detector, with a DB-5MS capillary column, 25 m × 0.32 mm i.d., 0.52 μm in film thickness). The oven temperature was programmed from 50 °C (3 min holding) to 290 °C at a rate of 10 °C/min and then held for 5 min. The injector temperature was maintained at 200 °C. When the authentic standard of germacrene D was analyzed with the injector temperature at 250 °C, some artificial peaks were observed. However, none of these peaks were detected when the injector was maintained at 200 °C. The analyses were replicated five times using different leaves for each species. The same analyses were performed on the leaf extractions collected in the field. 

Quantification analysis of germacrene D from greenhouse plants was performed with selected ion monitoring (SIM) using the ions *m/z* 161 (quantification ion) and *m/z* 91(qualifier ion). The mass spectrum of germacrene D is summarized as follows: *m/z* (%) 204 (M^+^, 17), 162 (14), 161 (100), 133 (18), 120 (21), 119 (32), 105 (51), 93 (20), 91 (42), 81 (27), 79 (26), 77 (21), 67 (10), 55 (11), 41 (18). The ions *m/z* 108 and 78 were used as qualification and qualifier ions for the internal standard (anisole), respectively. The ratio of peak area of qualification ion of germacrene D to internal standard was calculated. The concentration was determined by comparing the peak area ratio in the sample with those found in the calibration standard (2–85 ng/μL). 

## 4. Conclusions

The GC–MS analysis of the leaf extract of five *Bursera* revealed that they produce species-specific chemical components in their leaves. The study also shows the presence of germacrene D as a common sesquiterpene in the genus. 
